# TGF-β Signaling in the Pathophysiology of the Ovary: A Double-Edged Regulator

**DOI:** 10.3390/biom16010130

**Published:** 2026-01-12

**Authors:** Nicole Bertani, Alessandra Alteri, Luciana Cacciottola, Giorgia D’Addato, Gina La Sala, Biliana Lozanoska-Ochser, Micol Massimiani, Edoardo Parrella, Alessio Reggio, Eleonora Russo, Federica Campolo, Francesca Gioia Klinger

**Affiliations:** 1 Departmental Faculty of Medicine, UniCamillus-Saint Camillus International University of Health and Medical Sciences, 00131 Rome, Italyfederica.campolo@unicamillus.org (F.C.); 2 Faculty of Medicine and Surgery, University of Rome “Tor Vergata”, 00133 Rome, Italy; 3 Institute of Biochemistry and Cell Biology, Italian National Research Council, 00015 Monterotondo Scalo, Italy; 4 Department of Experimental Medicine, Sapienza University of Rome, Viale Regina Elena 324, 00161 Rome, Italy

**Keywords:** transforming growth factor-β, female fertility, folliculogenesis, ovarian diseases

## Abstract

The Transforming Growth Factor-β (TGF-β) superfamily comprises highly conserved cytokines that orchestrate key cellular functions, including proliferation, differentiation, and apoptosis. Within the ovary, TGF-β family members serve as pivotal regulators of folliculogenesis, exerting stage-specific actions from embryonic germ cell development to advanced follicular maturation. During fetal development, activins and SMAD-dependent signaling pathways are essential for primordial germ cell proliferation, survival, and the breakdown of germ cell cysts, enabling the establishment of the primordial follicle pool. Throughout folliculogenesis, TGF-β supports follicle activation, promotes the transition from dormant to growing follicles, stimulates granulosa cell proliferation, sustains follicular viability, and modulates steroidogenesis through theca cell regulation. Notably, anti-müllerian hormone, a TGF-β family member, plays a central role in inhibiting premature follicle recruitment and serves as a key biomarker of ovarian reserve. Dysregulation of TGF-β signaling contributes to various ovarian disorders, including polycystic ovary syndrome and premature ovarian insufficiency. A deeper understanding of these complex signaling networks is critical for identifying novel therapeutic targets and advancing clinical interventions in female reproductive pathologies. This review provides an integrated overview of the roles of the TGF-β superfamily in ovarian physiology and its contributions to disease development.

## 1. Introduction

The ovary is the central organ of the female reproductive system and its proper functioning is essential for reproductive success [1]. The ovarian follicles are the functional units of the ovary and are responsible for its two main functions: gametogenesis, which is related to the maturation and development of the female gametes, and endocrine function, which is associated with the production of steroid hormones, as well as other regulatory factors such as inhibins and activins. It is important to note that these functional outputs are embedded within the dynamic process of folliculogenesis. Folliculogenesis is characterized by a gradual and irreversible progression through different stages of follicles maturation. Maturing follicles are defined by size, differentiation, number, and organization of somatic cells that cooperate to sustain oocyte growth and developmental competence [2]. This process is finely regulated to ensure the sequential maturation of a cohort of follicles, starting from the primordial stage established during the fetal period.

The complex network of regulators that modulate the correct progression of folliculogenesis includes the transforming growth factor-β (TGF-β) superfamily, a large group of peptides with pleiotropic activities that are essential for homeostasis and morphogenesis in tissues of invertebrates and vertebrates [3]. The best-known members of this superfamily include bone morphogenetic proteins (BMPs), activins/inhibins, growth and differentiation factors (GDFs) and AMH. Their signaling pathway is mediated by type I and II serine/threonine kinase receptors, which, in turn, phosphorylate and activate transcription factors that enable the transcriptional activation of downstream target genes [4,5,6,7]. Within the ovary, TGF-β family members orchestrate key events throughout folliculogenesis. Accordingly, their signaling exerts stage-specific yet continuous regulatory actions that shape follicle development from the early stages to full maturation. From the embryonic stage onwards, TGF-β family members such as BMP and activin are needed for the assembly of primordial follicles and the formation of the ovarian reserve [8,9,10,11,12]. Subsequently, in early folliculogenesis, factors such as BMP15 and GDF9 enable cross-talk between the oocyte and the granulosa cells, which is essential for the correct activation and recruitment of follicles [13,14,15]. In later stages, TGF-β regulates sensitivity to gonadotropins, the expansion of the cumulus–oocyte complex (COC) and the regression of the corpus luteum [16,17]. The significant role of this family in regulating ovarian dynamics underscores its physiological relevance; however, alterations to these regulatory mechanisms can compromise follicular development and contribute to ovarian dysfunction and conditions such as polycystic ovary syndrome (PCOS) and premature ovarian insufficiency (POI) [18,19,20,21].

This review therefore aims to provide a comprehensive overview of the coordinated and integrated action of TGF-β superfamily members during folliculogenesis and ovarian dynamics. Specifically, it examines how tightly regulated TGF-β signaling exerts protective and homeostatic functions in ovarian development and follicle maturation. Conversely, its dysregulation contributes to pathological outcomes, including impaired folliculogenesis and ovarian disorders. This review will therefore examine and highlight the dual role of this superfamily as a regulator.

## 2. The TGF-β Superfamily: An Intricate Family Portrait

The TGF-β superfamily includes a large group of molecules capable of influencing cell fate decisions in almost every tissue of invertebrates and vertebrates [3]. In mammals, 33 different genes, both orthologues and paralogues, encode for factors that are strictly related to TGF-β [4]. Among them, the TGF-β subfamily, activins/inhibins, BMPs, AMH, glial-derived neurotrophic factors (GDNFs), and GDFs are particularly important in coordinating biological processes including embryonal development, postnatal tissue growth and homeostasis [4,22]. The complexity of this signaling system is further exacerbated by their ability to form homo- and heterodimers, by different receptor affinities and by the existence of antagonist factors, such as follistatin (FST), noggin (NOG), chordin (CHD), gremlin-1 (GREM1), and gremlin-2 (GREM2) [4]. All these superfamily cytokines are highly similar in terms of their modular domain structure [4,23]. In general, they are synthesized as inactive pre-proprotein monomers with a tripartite organization: a signal peptide, a large N-terminal pro-domain and a C-terminal, mature domain [4,24]. Dimers, such as homo- and heterodimers, are stabilized by a single inter-chain disulfide bond and hydrophobic interactions between two distinct monomers [25,26]. In this inactive form, the dimers is routed through the Golgi apparatus via the secretory pathway to undergo a dual processing step to complete the maturation process [26,27]. The first step occurs in the trans-Golgi network and consists of the pro-domain excision at the Arg-X-[Lys/Arg]-Arg consensus motif by furin convertases. Despite the cleavage, the pro-domain is not removed or degraded, but remains non-covalently bound to the carboxy-terminal domain [3,26,27]. In this seemingly buffered version imprinted by the presence of the N-terminal latency-associated peptide (LAP), the factor is now referred to as being in a “latent state” [25,28]. Ultimately, the cytokine activation can then occur by proteolytic removal of LAP by metalloproteinases, thus liberating the mature growth factor [29].

The core transduction system involves the ligand binding to the type II receptor, which then recruits and activates the type I receptor [4,6]. The activated receptor complex then phosphorylates the intracellular R-SMADs (SMAD2/3 for TGF-β/Activins; SMAD1/5/8 for BMPs/AMH) stimulating the formation of a heteromeric complex with the co-SMAD factor SMAD4 [6]. Ultimately, the SMAD complex translocates into the nucleus; recognizes a specific SMAD-binding element; and upon recruitment of additional co-factors, activates in-trans target gene expression [7,30]. Other cell-surface molecules, including β-Glycan (also known as the TGF-β type III receptor), can act as co-receptors for specific TGF-β superfamily ligands [31,32]. Although β-Glycan does not contain an intracellular signaling motif, it can bind TGF-β isoforms and inhibin by potentiating the presentation of the cytokines to type II receptors [23,31]. These “ligand trap” factors can be either secreted (e.g., follistatin, noggin, chordin and gremlin) or membrane-bound, operating by sequestering or increasing local ligand concentrations, depending on the cell and tissue context [7,33].

## 3. TGF-β Signaling in the Mammalian Ovary

In mammals, the TGF-β subfamily includes three ligands: TGF-β1, TGF-β2, and TGF-β3, all expressed in human and murine ovary [8,34,35]. TGF-β1 is expressed in granulosa cells (GCs), theca cells (TCs), and luteal cells, as well as in oocytes, whereas TGF-β2 is mainly expressed in granulosa and small luteal cells [36,37]. The third isoform, TGF-β3, is found to be expressed in oocytes of the fetal ovary [36,38]. Similarly, their receptor pairs are also expressed with a similar pattern in oocyte and ovary somatic cells [8,36,37]. While the expression of these factors seems to be consistent in these cell types across ovulating species, considerable variations are evident in the spatio-temporal expression pattern of individual isoforms during development or in the physiological ovary dynamics [39].

The signaling network is crucial for ovarian physiology; the TGF-β subfamily coordinates multiple dynamics occurring in the ovary, including follicle growth, GCs proliferation and ovulation [39]. The trophic activity of these autocrine/paracrine factors influences adjacent stromal cells and oocytes [34]. TGF-β ligands produced by GCs are essential regulators of oocyte meiosis, folliculogenesis, and stromal remodeling [16]. In this physiological context, tightly regulated TGF-β signaling plays a protective and homeostatic role, ensuring the proper development of follicles and the tissue organization. The loss of signaling components, such as SMAD2 and 3, has been shown to have a substantial impact on female fertility, leading to impaired ovarian function [40]. Furthermore, abnormal levels of TGF-β ligands or increased cell sensitivity to these factors were associated with follicular dysplasia and ovarian functional failure [18,19], conditions that frequently coincide with the development of ovarian fibrosis [18,20]. The precise regulation of TGF-β levels in both time and space is essential for maintaining optimal ovarian homeostasis. Evidence from in vitro studies supports the notion that TGF-β signaling also exerts concentration and exposure dependent effects on ovarian cells. While low or tightly regulated levels of TGF-β activity promote granulosa cell survival, differentiation and follicle support, higher or sustained levels have been shown to inhibit proliferation and induce apoptotic or profibrotic responses, particularly within the stromal compartment [18,19,20]. These observations emphasize the dual nature of TGF-β signaling in the ovary, whereby it acts as a “double-edged regulator” whose beneficial effects depend on precise temporal, spatial and quantitative control.

While the role of TGF-β signaling through canonical SMAD-dependent mechanisms in ovarian physiology is well established, the contribution of SMAD-independent pathways remains poorly defined. In non-ovarian compartments, such as fibroblasts [41] and human retinal pigment epithelial cells [42], TGF-β has been shown to activate non-canonical signaling cascades, including MAPK and PI3K/AKT pathways. These pathways are known regulators of primordial follicle activation and granulosa cell fate; however, how they are integrated into TGF-β signaling within ovarian tissue is not yet fully understood. In this context, Wang et al. (2014) demonstrated that TGF-β does not modulate the PI3K/AKT pathway during follicular activation, but instead affects the TSC–mTORC1 axis [43], highlighting a potential divergence between ovarian and non-ovarian signaling mechanisms. Together, these observations indicate that significant gaps remain in the understanding of SMAD-independent TGF-β signaling in the ovary, particularly regarding whether non-canonical pathways act in parallel, synergistically, or independently from canonical SMAD signaling in ovarian cells. Clarifying the contribution of pathways such as MAPK and PI3K/AKT may therefore be relevant for interpreting the context-dependent effects of TGF-β on follicle activation, granulosa cell behavior, and ovarian stromal remodeling. Given this complex and pleiotropic regulatory role, the specific actions of the TGF-β subfamily and other related factors—such as Activins, Inhibins, GDF9, and BMPs—will be discussed in detail in the following dedicated sections.

## 4. Role of TGF-β in Fetal Ovary Development

The first evidence of TGF-β signaling in the ovary was provided by Feng and colleagues in the late 1980s [44]. They showed that TGF-β accelerates meiotic maturation in rat oocytes by promoting germinal vesicle breakdown through cumulus-cell-mediated mechanisms [44]. Immunohistochemical analyses of human fetal ovaries demonstrated the differential expression of TGF-β1, TGF-β2, and TGF-β3, together with their receptors, across germ and somatic cell compartments, suggesting key morphogenetic functions in stromal patterning and primordial follicle assembly [8]. These observations were corroborated by evidence obtained in bovine fetal ovaries, where extracellular-matrix-associated regulators such as fibrillins and latent transforming growth factor-β binding proteins (LTBPs) control ligand bioavailability, thereby generating gradients essential for follicle assembly and somatic cell differentiation [45].

**Figure 1:**
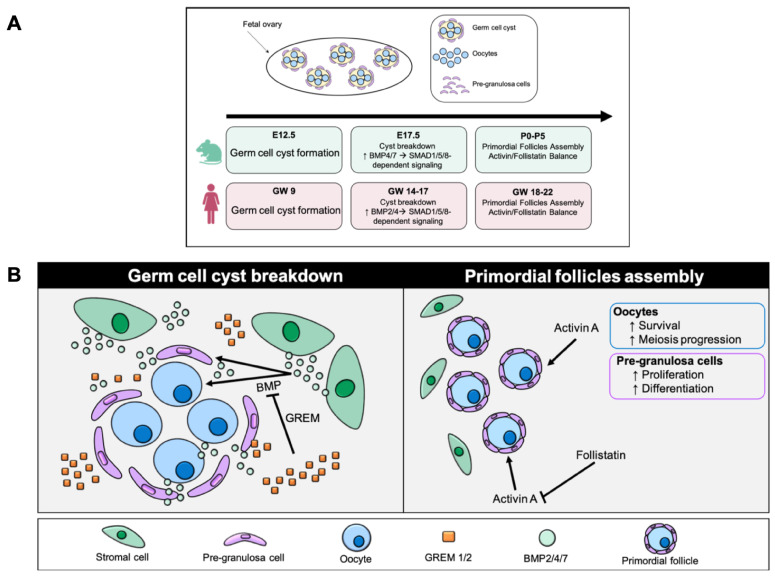
TGF-β in fetal ovary development. (**A**) Comparative timeline of TGF-β family signaling during fetal ovary development in mouse and human. This diagram shows the main stages in the formation of germ cyst, their breakdown and the assembly of primordial follicles in two species. In mice (E12.5–P5) and humans (GW 9–22), germ cell cyst breakdown is associated with increased BMP2/4/7–SMAD1/5/8/4 activity, while follicular assembly depends on the balance between activin A and follistatin (E = embryonic day in mice; P = postnatal day in mice; GW = gestational week in humans). (**B**) The left panel illustrates the phase of germ cell cyst breakdown, during which oocytes initially organized in interconnected cysts become progressively individualized. BMP signals (primarily BMP2/4/7), produced by the ovarian stroma, promote cyst dissociation and oocyte individualization, facilitating the transition toward primordial follicle formation. GREM antagonists (GREM1/2) restrict BMP activity by modulating the magnitude and spatial distribution of the signal, thereby preventing premature or excessive follicular activation. The right panel illustrates primordial follicle assembly, a stage in which each oocyte is surrounded by pre-granulosa cells. Activin A supports oocyte survival, promotes meiotic progression, and stimulates proliferation and differentiation of pre-granulosa cells, ensuring proper initiation of folliculogenesis. Follistatin, an antagonist of activin A, modulates its activity to maintain the balance between somatic cell proliferation and follicle maturation. The activin–follistatin axis thus ensures the structural and functional formation of primordial follicles.

Studies on rodent models further confirmed that members of the TGF-β pathway are dynamically expressed throughout gestation, with peaks coinciding with germ cell nest breakdown and follicle formation (Figure 1A) [46]. In mice, BMP4 and BMP7 produced by ovarian stromal cells stimulate the transition of germ cell cysts to primordial follicles through SMAD1/5/8-dependent signaling [9] whereas in the human fetal ovary, BMP2 and BMP4 expression coincides with the onset of follicle formation, while antagonists such as GREM1 and GREM2 spatially restrict signaling, preventing excessive follicular activation (Figure 1B) [47]. Oocyte-derived BMP15 and GDF9, first described in rodents and later confirmed in bovine and human ovaries, mediate bidirectional communication with pre-granulosa cells to regulate proliferation, gap junction formation, and oocyte growth [48,49,50]. Knockout mouse models of TGF-β signaling pathway members have been essential in uncovering the functional roles of this signaling network in embryonic folliculogenesis, confirming its critical involvement in germ–somatic cross-talk, oocyte survival, and follicle assembly. Mice lacking GDF9 exhibit follicular arrest at the primary stage, while Bmp15-null animals display defective follicle growth and ovulatory failure [51,52]. In humans, GDF9 is transiently expressed in oocytes before follicle formation and regulated by NOBOX, which is essential for establishing oocyte competence [49]. This expression pattern is similar across different species, particularly in bovine models, indicating a conserved oocyte–somatic feedback mechanism [46]. Activins and Inhibins form a complementary axis within the TGF-β family, fine-tuning germ cell proliferation and follicular organization. Activin A and its receptors are expressed in both germ and somatic cells of both human and mouse fetal ovaries, promoting germ cell survival, proliferation, and meiotic progression [10,11,12]. In vitro exposure of human fetal ovarian tissue to activin A enhances germ cell proliferation while suppressing apoptosis prior to follicle enclosure [53]. Conversely, follistatin and inhibins antagonize activin signaling after follicle formation, preventing excessive proliferation and ensuring structural integrity (Figure 1B) [54]. In both human and bovine ovaries, follistatin transcription is co-regulated by FOXL2 and BMP2, integrating BMP and activin feedback loops [55]. It is interesting to note that mouse models lacking activin signaling members show impaired follicle assembly and defective oocyte maturation, confirming the need for a balanced activin–follistatin axis [56]. The coordination between BMP- and activin-mediated signaling relies on canonical SMAD pathways. SMAD1/5 activation (BMP arm) and SMAD2/3 activation (activin/TGF-β arm) jointly regulate germ–somatic equilibrium, balancing proliferation and apoptosis [53,57]. Disruption of these signaling branches in murine models leads to altered follicle numbers and aberrant GCs differentiation [58]. In bovine fetal ovaries, differential SMAD activation correlates with follicle density and stromal organization, suggesting that these pathways define the final size of the ovarian reserve [45]. At the tissue level, TGF-β1 modulates extracellular matrix remodeling and stromal collagen deposition in fetal rat ovaries, linking morphogenesis to follicle assembly [59]. In this context, a tight integration with MAPK and PI3K signaling ensures context-specific responses, while crosstalk with WNT/β-catenin and p53 pathways provides fine temporal control [60,61]. In addition to intrinsic genetic regulation, the TGF-β signaling network operating during ovarian development is particularly vulnerable to environmental and pharmacological influences that can modulate its expression and downstream effects. Environmental and pharmacological factors can perturb these tightly regulated pathways. Prenatal exposure to acetaminophen or amoxicillin in mice and humans alters the expression of TGF-β-related genes, leading to reduced germ cell numbers and abnormal follicle formation [62,63]. High intra-ovarian steroid levels can further disrupt primordial follicle assembly interfering with TGF-β-mediated extracellular remodeling [64]. Such findings emphasize the sensitivity of the TGF-β network to hormonal and xenobiotic influences, with implications for the establishment of the ovarian reserve. Overall, evidence from human, mouse, rat, and bovine studies indicates that embryonic folliculogenesis is governed by a multifaceted TGF-β signaling network. This network integrates BMP, activin, and GDF inputs through both SMAD-dependent and SMAD-independent mechanisms that regulate oocyte survival, GCs differentiation and extracellular matrix organization. The precise temporal and spatial regulation of TGF-β family members ensures the proper assembly of primordial follicles, the foundation of postnatal ovarian function and lifelong fertility.

## 5. TGF-β Signaling in Early Folliculogenesis

**Figure 2:**
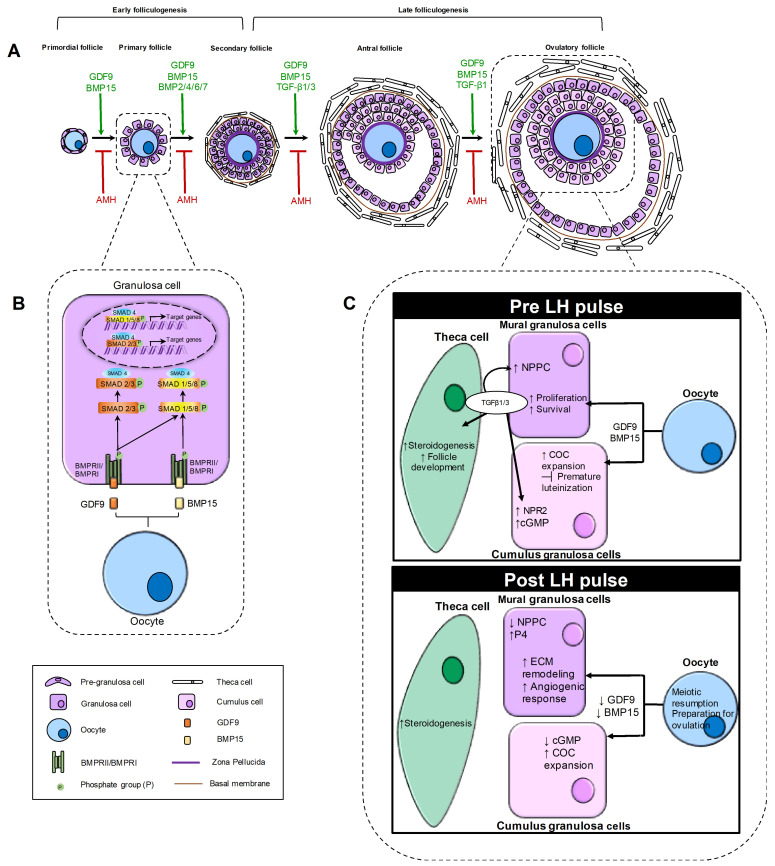
Schematic representation of the role of TGF-β family in ovarian folliculogenesis. The figure illustrates the dynamic role of TGF-β superfamily members throughout the progression of ovarian follicles from the primordial to the ovulatory stage. (**A**) Follicular progression and key TGF-β ligands involved. The figure illustrates the sequential stages of folliculogenesis, showing the progression of ovarian follicles from the primordial to the ovulatory stage. For each stage, it highlights the TGF-β family members that promote follicular growth (shown in green), as well as the inhibitory factors (shown in red) that act during both early and late folliculogenesis. (**B**) TGF-β signaling pathway in early folliculogenesis. The binding of GDF9 and BMP15 ligands produced by the oocyte to type I and II receptors expressed on the GC membrane induces the phosphorylation of specific R-SMADs (SMAD1/5/8 and SMAD2/3). The phosphorylated R-SMADs form a complex with SMAD4 and translocate to the nucleus to activate or repress genes involved in granulosa cell differentiation and proliferation. (**C**) TGF-β signaling pathway in late folliculogenesis. The panels compare TGF-β signaling activity in mural granulosa and cumulus cells under pre-LH and post-LH pulses. In the pre-LH pulse condition, the oocyte produces GDF9 and BMP15, acting on both mural granulosa and cumulus cells. In mural granulosa cells, they promote proliferation and survival, while in cumulus cells they support COC expansion and inhibit premature luteinization. Mural granulosa cells also produce TGF-β1/3, which act autocrinely to increase NPPC and paracrinely on cumulus cells to upregulate NPR2 and cGMP. In theca cells, TGF-β1/3 enhances steroidogenesis. In the post-LH pulse condition, oocyte production of GDF9 and BMP15 decreases. In mural granulosa cells, this reduction is associated with extracellular matrix (ECM) remodeling, increased angiogenesis and decreased NPPC production, as well as increased progesterone (P4) levels. The decline in GDF9 and BMP15 also reduces cGMP levels, which stimulates meiotic resumption in the oocyte. In theca cells, steroidogenesis is promoted during this phase.

Early folliculogenesis represents the initial phase of follicular development, encompassing the transition from primordial to secondary follicles. Although this stage is thought to be independent of gonadotropin action [65], it is finely orchestrated by intraovarian signaling networks, among which members of the TGF-β superfamily play pivotal roles (Figure 2A). The activation of quiescent primordial follicles is a key event determining the size and longevity of the ovarian reserve and depends on paracrine communication between the oocyte and pre-granulosa cells [13]. Upon activation, morphological changes occur in both the pre-granulosa cells and the oocyte, where the oocyte increases in diameter [66], and simultaneously, pre-granulosa cells proliferate while undergoing morphological changes to adopt a cuboidal phenotype [67]. A transitional phase is observed where the follicle contains both flat, squamous pre-granulosa cells typical of primordial follicles and a certain number of cuboidal GCs, which eventually replace the first one, leading to the primary follicle stage.

In this context, oocyte-derived growth factors of the TGF-β family act as central regulators of follicle activation and progression. Within this group, GDF9 is known to be highly expressed in oocytes and, to a lesser extent, in granulosa-luteal cells, suggesting it plays important roles in both cell types [68,69]. Similar to other TGF-β superfamily ligands, GDF9 initiates signaling by binding to type I and type II (BMPRII) receptors with serine/threonine kinase activity, followed by the phosphorylation of intracellular SMAD (SMAD1/5/8) transcription factors [7]. Adult homozygous GDF9 null female mice exhibit sterility, with significantly smaller ovaries and compromised folliculogenesis, where primary follicles do not transition to secondary follicles [68,70]. Although oocytes in the primary follicles of GDF9 null mice grow at a rate similar to that of wild-type mice, cytological abnormalities become apparent and the oocytes undergo progressive cell death. These cytological abnormalities include aggregation of perinuclear organelles, unusual peripheral Golgi complexes and failure to form cortical granules [68,69]. Similarly, studies on rats indicate that GDF-9 stimulates the growth of primary follicles [14,71]. In vitro studies using ovarian fragments from women have shown that GDF-9 can support the survival and growth of oocytes, as well as follicular maturation, thereby promoting the transition of primordial follicles to secondary follicles [14]. These studies suggest a conserved role for GDF9 in various mammalian species.

BMPs were initially identified for their osteogenic properties [33], also contributes significantly to early folliculogenesis. BMPs bind to their heteromeric transmembrane serine/threonine kinase receptors, BMPR-1 and BMPR-2 [72]. Specifically, binding of BMP to BMPR-2 promotes the recruitment and activation of BMPR-1, which then phosphorylates SMAD proteins that form a heteromeric complex with SMAD4. This complex then moves to the nucleus to stimulate the gene expression of various target genes (Figure 2B) [7]. Several BMPs are expressed within the ovary, including BMP-2, 4, 6 and 7, which regulate folliculogenesis, oocyte growth, luteinization and ovarian steroidogenesis [72]. However, BMP15 plays a fundamental role in the early stages of folliculogenesis by promoting the growth and maturation of early follicles in conjunction with GDF9 [73]. During primordial follicle activation, communication between GCs and oocytes induces the latter to produce BMP15 [15]. The BMP15 ligand then binds to the BMPRII receptor, which is expressed on GCs, promoting the phosphorylation of SMAD1/5/8 and the formation of a complex with SMAD4. This complex then translocates to the nucleus, where it regulates the transcription of Kit-Ligand and other genes involved in the proliferation and differentiation of GCs [50]. Therefore, the activation of primordial follicles is tightly regulated by oocyte-derived TGF-β ligands, mainly GDF9 and BMP15, which coordinate intracellular signaling and oocyte–somatic cell communication to preserve the ovarian reserve and sustain female fertility.

## 6. TGF-β Family Regulator Bridging Early and Late Folliculogenesis

Once recruited and activated, the primordial follicle continues to grow toward the subsequent stages of maturation. Primary to secondary transition of growing follicles is characterized by oocyte enlargement and maturation, accompanied by secretion of the zona pellucida, a glycoprotein-rich membrane essential for sperm binding and fertilization [74]. During this phase, GCs actively proliferate, forming multiple layers around the growing oocyte (reaching a total follicular diameter of approximately 120–200 µm), while TCs are recruited from the ovarian stroma. These TCs differentiate into an inner steroidogenic layer, producing androgens later aromatized into estrogens by GCs, and an outer structural and vascular layer [75]. At this stage, follicle development remains gonadotropin-independent, although follicle-stimulating hormone (FSH) progressively increases GC sensitivity [76].

To preserve the ovarian reserve, the pace of follicle recruitment and growth is tightly controlled. The inhibitory regulation is essential to prevent primordial follicle burnout and is mediated by AMH, a TGF-β family member secreted by GCs of secondary and small antral follicles. In rodents, AMH has long been known to suppress primordial follicle activation (Figure 2A) [77] through paracrine action on anti-müllerian hormone type-2 receptor (AMHR2) expressed in GCs. It inhibits primordial follicle activation by preventing GC recruitment and decreasing their responsiveness to stimulatory growth factors such as basic fibroblast growth factor (bFGF) and kit ligand (KITL), which are known activators of primordial follicle growth [78]. The ability of AMH to modulate early folliculogenesis has been explored to regulate follicle dynamics in vitro [79] and in pathological contexts such as gonadotoxic injury, where excessive follicular activation leads to accelerated depletion [80,81]. However, evidence of similar effects in monovulatory species such as humans remains less clear. AMH expression has been reported in primordial follicles of primates [82] and humans [83], but its ability to effectively block primordial activation is still not fully elucidated. Early in vitro studies showed discrepant outcomes depending on AMH dosage in human ovarian tissue culture models [84,85]. These inconsistencies may derive from differences in administration methods (including hormone penetration into the tissue) and variability in recombinant AMH preparations with potentially distinct bioactivity [86,87,88]. More recent studies have reinforced the hypothesis of an AMH direct inhibitory role, particularly in non-physiological conditions associated with aberrant follicle activation, resulting in improved follicle survival, as observed in xenotransplantation models of human ovarian injury [89,90] and in prolonged in vitro culture [91]. Furthermore, AMH exerts an inhibitory effect on growing preantral follicles, regulating their transition into the gonadotropin-dependent stage. Specifically, AMH signaling modulates GC function through several mechanisms: it reduces GC responsiveness to FSH and inhibin B by downregulating their respective receptors [92,93], reduces estradiol synthesis by suppressing aromatase expression [94,95] and influences GC proliferation rates and apoptosis in both in vitro and in vivo models [96]. Within the early antral follicular cohort, the inhibitory actions of AMH serve to prevent premature follicle selection. Dominance occurs only when a single follicle progressively lowers its own AMH production, thereby escaping this inhibition and gaining a growth advantage over the remaining cohort.

## 7. TGF-β Signaling in Late Folliculogenesis

During the transition from secondary to antral follicles, a small cavity called an “antrum” begins to form gradually inside the follicle. During this phase, the follicles become increasingly reactive and dependent on gonadotropins, and two types of GCs can be distinguished within the antral follicle: the mural granulosa cells, responsible for hormone production, and the cumulus cells, which surround the oocyte [2]. Even at this stage of folliculogenesis, TGF-β signaling exerts a multifaceted regulatory role, coordinating the interaction between granulosa, theca, and oocyte compartments to sustain follicle growth, steroidogenesis, and oocyte competence. Both TGF-β1 and TGF-β3 are highly expressed in mural granulosa cells of large antral follicles, while their receptors (TGFBR1/TGFBR2) are abundant in TCs and cumulus cells [16]. Recent studies have identified specific mechanisms by which TGF-β1 and TGF-β3, through activation of TGFBR1/TGFBR2 and the downstream SMAD2/3-SMAD4 pathway, regulate key GCs functions [97,98]. In particular, TGF-β signaling enhances transcription of natriuretic peptide type C (NPPC) secreted by mural granulosa cells, maintaining oocyte meiotic arrest via its cognate receptor natriuretic peptide receptor 2 (NPR2), which produces cyclic guanosine monophosphate (cGMP). Conditional deletion of *Tgfbr2* in these cells disrupts NPPC production, impairs antral follicle development, and compromises fertility, underscoring the physiological relevance of this pathway [99]. As follicles approach the pre-ovulatory phase, the LH surge induces a transient down-regulation of TGFBR2 and a reduction in SMAD2/3 phosphorylation in mural granulosa cells, which alleviates the inhibitory tone of TGF-β and permits LH-driven differentiation and luteinization [97,98]. During the peri-ovulatory stage, TGF-β family members, including oocyte-derived GDF9 and BMP15, act synergistically with gonadotropin-induced EGF-like peptides to drive COC expansion and oocyte maturation (Figure 2C) [17]. These factors activate SMAD2/3 or SMAD1/5/8 signaling in cumulus cells, inducing the expression of matrix-associated genes such as HAS2, PTGS2, PTX3, and TNFAIP6, which are essential for hyaluronic acid synthesis and extracellular matrix stabilization during cumulus expansion. Oocyte removal (oocytectomy) results in impaired cumulus expansion and premature luteinization, effects reversed by supplementation with oocyte-secreted factors or recombinant GDF-9/BMP-15 [17,100]. In addition to promoting COC expansion, TGF-β family ligands exert anti-luteinizing and anti-apoptotic actions on granulosa and cumulus cells. Oocyte-derived GDF9 and BMP15 suppress FSH-induced expression of LHCGR and CYP11A1, preventing premature progesterone production and maintaining cumulus identity [17,101,102]. These factors also promote GCs proliferation and inhibit apoptosis by upregulating Bcl-2 and downregulating Bax and caspase-3, preserving follicular integrity and oocyte competence [103]. During ovulation and luteinization, TGF-β1 and its downstream SMAD2/3 signaling in mural granulosa cells facilitate extracellular matrix remodeling and angiogenic responses that accompany corpus luteum formation, while BMP-15 and GDF-9 gradually decline in expression, allowing the luteinization program to proceed (Figure 2C) [16,104]. Conversely, in follicles destined for atresia, dysregulation of TGF-β signaling contributes to apoptotic degeneration. Chu et al. (2018) demonstrated that both SMAD-dependent and non-SMAD-dependent pathways of the TGF-β superfamily influence granulosa cell apoptosis to determine cell survival [105]. As the luteal phase progresses, the role of TGF-β shifts toward regression and fibrosis. In luteolysis, prostaglandin F_2_α (PGF_2_α) triggers a cascade of vasoactive and inflammatory mediators, including TNF-α, thrombospondin-1 (THBS1), and TGF-β1, that promote endothelial cell apoptosis and microvascular regression [106]. TGF-β1 is upregulated in regressing corpora lutea and acts synergistically with THBS1 to induce capillary destabilization and fibrotic remodeling through SMAD-dependent pathways [107,108].

## 8. TGF-β Pathway Dysregulation in Ovarian Disorders: A Focus on POI and PCOS

Under physiological conditions, as it is described above, TGF-β signaling plays a key role in maintaining ovarian homeostasis by supporting the coordinated growth of follicles, the survival of granulosa cells, the controlled remodeling of the stroma and the timely progression through the stages of folliculogenesis. The precise regulation of TGF ligands and downstream signaling ensures the preservation of the ovarian reserve and the proper maturation of follicles. Importantly, TGF-β signaling does not operate in isolation in vivo but is embedded within a complex ovarian micro-environment in which hormones and cytokines critically shape its functional outcome. Gonadotropin signaling intersects with TGF-β pathways to fine-tune follicular maturation, and the LH surge induces a transient attenuation of TGF-β/SMAD2-3 activity, enabling ovulation and luteal differentiation [97,99]. In parallel, inflammatory cytokines such as TNF-α, which are increased during luteolysis and in pathological conditions including PCOS, can synergize with TGF-β signaling to promote granulosa cell apoptosis and stromal remodeling [105,109]. AMH further exemplifies this integrated regulation by modulating follicular sensitivity to gonadotropins and restrain follicle recruitment [77,95]. When this finely tuned regulatory balance is disrupted, TGF-β signaling shifts from a homeostatic and protective role to driving ovarian pathophysiology, contributing to abnormal follicular dynamics and tissue remodeling. Ovarian stromal fibroblasts and perivascular cells play a central role in orchestrating cyclical tissue remodeling associated with follicular growth and ovulation. These cell populations respond to tightly regulated TGF-β signaling to coordinate extracellular matrix turnover and vascular remodeling [110]. However, when TGF-β activity becomes excessive or insufficiently counterbalanced, such as in the context of chronic inflammation or pathological states like ovarian endometriosis [111], this pathway may shift toward a profibrotic program. Indeed, activation of the TGF-β/SMAD pathway in these cells induces extracellular matrix deposition, specifically upregulating collagen I/III and fibronectin, and reduced matrix degradation through downregulation of MMP 2/9/13 activity [112]. TGF-β also promotes the differentiation of stromal fibroblasts into α-SMA-positive myofibroblasts, through ACTA2 gene expression, contributing to vascular remodeling and structural reinforcement of the ovarian stroma [113].

POI and PCOS are two ovarian disorders associated with defects in folliculogenesis that exhibit distinct etiologies and clinical presentations associated with dysregulation of TGF-β signaling and follicle development abnormalities. POI affects approximately 1% of women under 40 and 0.1% of those under 30 [114,115]. It is defined by declining ovarian function, low estradiol levels, elevated gonadotropins (FSH > 40 IU/L), and oligomenorrhea or amenorrhea lasting more than four months [107]. Its etiologies are heterogeneous and include genetic abnormalities, autoimmune dysfunction, iatrogenic ovarian damage, metabolic or infectious conditions, and idiopathic mechanisms leading to accelerated follicular depletion or dysfunction [115]. As no treatment can currently restore ovarian function, hormone replacement therapy (HRT) remains the first-line approach to manage POI-related clinical consequences [116]. Several members of the TGF-β superfamily are dysregulated in POI, including BMP15, GDF9, and TGF-β/Smad3, resulting in impaired folliculogenesis (BMP15, GDF9) and excessive stromal remodeling with fibrosis (TGF-β/Smad3) [2]. Genetic variants in BMP15 and GDF9 are highly prevalent among women with a POI phenotype [117]. Notably, the BMP15 V136L mutation reduces BMP15 transcriptional activity in granulosa cells, contributing to the POI phenotype [118]. Overall, POI arises from the interplay between genetic predisposition and ovarian stressors, with TGF-β signaling at the core of its pathogenesis, highlighting the dual contribution of intrinsic and extrinsic factors and pointing to TGF-β pathways as potential therapeutic targets.

PCOS is a different but equally important example of how TGF-β dysregulation can cause follicular arrest and other ovarian changes. PCOS is a heterogeneous endocrine-metabolic disorder affecting 4–20% of women of reproductive age and is a leading cause of anovulatory infertility [119]. It is characterized by hyperandrogenism, ovulatory dysfunction, and polycystic ovarian morphology, often accompanied by insulin resistance, low-grade inflammation, and increased ovarian fibrosis [120]. Neuroendocrine dysfunction in PCOS includes increased GnRH pulse frequency, leading to elevated LH secretion and relative preservation or suppression of FSH, reflecting reduced sensitivity of the hypothalamic–pituitary axis to steroid feedback [121]. Excess LH promotes androgen production in TCs, whereas reduced FSH impairs GCs function and follicular maturation, contributing to anovulation [122]. Abnormalities in the TGF-β pathway have been implicated in the multifactorial pathogenesis of PCOS, affecting ovarian structure, follicular development, and metabolic homeostasis. Dysregulation of several pathway members—such as follistatin, fibrillin, activin, inhibin, AMH, BMP, and GDF9—has been associated with excessive stromal proliferation, extracellular matrix deposition, and follicular arrest [123,124,125,126,127]. Women with PCOS exhibit higher circulating follistatin and lower activin A levels, with no differences in inhibin B. Given the stimulatory role of activins and the inhibitory effects of inhibins and FST on FSH release [128], this imbalance may hinder follicular maturation beyond 8–10 mm, contributing to the absence of pre-ovulatory follicles [129]. Fibrillin 3 (FBN3) has also been linked to PCOS. A variant in the D19S884 dinucleotide repeat marker (allele 8), located within intron 55 of FBN3, is associated with increased fasting insulin levels in women with PCOS and their brothers [130]. Raja-Khan and colleagues further demonstrated that carriers of allele 8 have lower total TGF-β1 but higher inhibin B and aldosterone, highlighting an association between FBN3 polymorphisms and alterations in TGF-β signaling [131]. More recent evidence reports elevated TGF-β1 expression and altered SMAD2/3 activation in GCs from PCOS ovaries, potentially impairing follicle development by increasing granulosa cell apoptosis [109]. Although earlier studies described dysregulation of several TGF-β superfamily members, such as reduced GDF9 [132], altered AMH levels [126,133], and increased AMHR2 [134], a more recent genetic analysis found no significant associations between variants in GDF9, BMP15, AMH, or AMHR2 and PCOS susceptibility, though some GDF9 variants were linked to hirsutism scores and parity, suggesting modifier rather than causal roles [127]. Together, these findings emphasize the role of TGF-β signaling disruptions in the pathological trajectories of both POI and PCOS.

## 9. Conclusions

This review highlights the TGF-β superfamily as an essential regulator of ovarian functions orchestrating folliculogenesis from primordial follicle assembly to ovulation and luteal remodeling. Within the ovary, members of this family finely coordinate the proliferation and differentiation of somatic cells, oocyte survival and growth, and bidirectional communication between oocyte and GCs, as well as the regulation of steroidogenesis through complex SMAD-dependent and SMAD-independent pathways. A key emerging concept is the dual nature of TGF-β signaling as a “double-edged regulator” of ovarian function. Under physiological conditions, balanced and stage-specific activation of TGF-β family members, such as GDF9, BMP15, AMH, activins and BMPs, plays a protective role by preserving the ovarian reserve, ensuring orderly follicle recruitment, supporting oocyte competence and enabling the selection and luteinization of the dominant follicle. Conversely, when this finely tuned signaling network is disrupted, the same pathways can become pathogenic, contributing to follicular dysfunction, impaired maturation, stromal remodelling and fibrosis. This duality is particularly evident in ovarian disorders such as POI and PCOS, where dysregulated TGF-β signaling is associated with accelerated follicle depletion, excessive stromal fibrosis, granulosa cell apoptosis, and follicular arrest. While this review outlines significant findings, there are still crucial knowledge gaps. In particular, the contribution of SMAD-independent TGF-β signaling pathways to ovarian physiology and pathology remains unclear, as is the integration of TGF-β signals with other key regulatory networks that control folliculogenesis. Furthermore, the dynamic and stage-specific balance between TGF-β family ligands, their receptors and extracellular antagonists, such as AMH, follistatin and BMP inhibitors, remains poorly understood. Disruption of this balance appears to drive the progression from protective, physiological signalling to pathological outcomes. Future research should therefore focus on clarifying the temporal and cell-specific regulation of TGF-β signalling, balancing its protective and deleterious effects, and defining how these pathways are altered in disease states. Advancing this knowledge is essential for identifying novel targets and developing strategies that can selectively modulate TGF-β signalling. The overarching goal is to preserve ovarian function and improve the management of female reproductive disorders.

## Data Availability

No new data were created or analyzed in this study.

## Abbreviations

The following abbreviations are used in this manuscript:

TGF-β	Transforming Growth Factor-beta
BMPs	Bone Morphogenetic Proteins
AMH	Anti-Müllerian Hormone
GDNFs	Glial-Derived Neurotrophic Factors
GDFs	Growth and Differentiation Factors
FST	Follistatin
NOG	Noggin
CHD	Chordin
GREM1	Gremlin-1
GREM2	Gremlin-2
LAP	Latency-Associated Peptide
R-SMADs	Receptor-regulated SMADs
GCs	Granulosa Cells
LTBPs	Latent Transforming Growth Factor-beta Binding Proteins
MAPK	Mitogen-Activated Protein Kinase
PI3K	Phosphoinositide 3-Kinase
WNT	Wingless-related integration site
GDF9	Growth Differentiation Factor 9
BMP15	Bone Morphogenetic Protein 15
TCs	Theca Cells
FSH	Follicle-Stimulating Hormone
AMHR2	Anti-Müllerian hormone type-2 receptor
bFGF	Basic Fibroblast Growth Factor
KITL	Kit Ligand
LH	Luteinizing Hormone
NPPC	Natriuretic Peptide Type C
NPR2	Natriuretic Peptide Receptor 2
COC	Cumulus–Oocyte Complex
HAS2	Hyaluronan Synthase 2
PTGS2	Prostaglandin-Endoperoxide Synthase 2
PTX3	Pentraxin 3
TNFAIP6	TNF Alpha Induced Protein 6
LHCGR	Luteinizing Hormone/Choriogonadotropin Receptor
CYP11A1	Cytochrome P450 Family 11 Subfamily A Member 1
PGF_2_α	Prostaglandin F_2_ α
HRT	Hormone Replacement Therapy
FBN3	Fibrillin 3
